# Addressing loneliness and social isolation in 52 countries: a scoping review of National policies

**DOI:** 10.1186/s12889-024-18370-8

**Published:** 2024-05-01

**Authors:** Nina Goldman, Devi Khanna, Marie Line El Asmar, Pamela Qualter, Austen El-Osta

**Affiliations:** 1 Manchester Institute of Education, University of Manchester, Ellen Wilkinson Building, Devas Street, Manchester, M13 9PL United Kingdom; 2 School of Public Heath, Faculty of Medicine, Imperial College London, Charing Cross Hospital, Reynolds Building, St Dunstan’s Road, London, W6 8RF United Kingdom; 3 North Hampshire Hospital, Hampshire Hospitals NHS Foundation Trust, Basingstoke, United Kingdom

**Keywords:** Loneliness, Social isolation, Policy analysis, Europe, Review, Policy recommendation

## Abstract

**Background:**

Even prior to the advent of the COVID-19 pandemic, there was ample evidence that loneliness and social isolation negatively impacted physical and mental health, employability, and are a financial burden on the state. In response, there has been significant policy-level attention on tackling loneliness. The objective of this scoping review was to conduct a loneliness policy landscape analysis across 52 countries of the UN European country groups. Our policy analysis sought to highlight commonalities and differences between the different national approaches to manage loneliness, with the goal to provide actionable recommendations for the consideration of policymakers wishing to develop, expand or review existing loneliness policies.

**Methods:**

We searched governmental websites using the Google search engine for publicly available documents related to loneliness and social isolation. Seventy-eight documents were identified in total, from which 23 documents were retained. Exclusion of documents was based on predetermined criteria. A structured content analysis approach was used to capture key information from the policy documents. Contextual data were captured in a configuration matrix to highlight common and unique themes.

**Results:**

We could show that most policies describe loneliness as a phenomenon that was addressed to varying degrees in different domains such as social, health, geographical, economic and political. Limited evidence was found regarding funding for suggested interventions. We synthesised actionable recommendations for the consideration of policy makers focusing on the use of language, prioritisation of interventions, revisiting previous campaigns, sharing best practice across borders, setting out a vision, evaluating interventions, and the need for the rapid and sustainable scalability of interventions.

**Conclusions:**

Our study provides the first overview of the national loneliness policy landscape, highlighting the increasing prioritisation of loneliness and social isolation as a major public health and societal issue. Our findings suggest that policymakers can sustain this momentum and strengthen their strategies by incorporating rigorous, evidence-based intervention evaluations and fostering international collaborations for knowledge sharing. We believe that policymakers can more effectively address loneliness by directing funds to develop and implement interventions that impact the individual, the community and society.

**Supplementary Information:**

The online version contains supplementary material available at 10.1186/s12889-024-18370-8.

## Introduction

The significant increase in research on loneliness and social isolation over the last decade, and especially following the advent of the COVID-19 pandemic [1–3] highlighted the detrimental consequences of loneliness to individuals, society and governments worldwide. For older adults, the pandemic led to feelings of loneliness due to a lack of companionship and connections, which can negatively impact cognition, and mental health [4]. The paradox of social distancing, intended to protect older adults, further isolated them and exacerbated the negative effects of loneliness [5]. A longitudinal study on adolescents showed that they also experienced social isolation from peers, and that resulted in increases of loneliness due to COVID-related school closures [6]. Evidence shows that a lack of social connection impacts physical and mental health [7], employability opportunities [8], and how it is related to social disparities [1, 9]. In response, there has been significant policy-level attention on loneliness, with, for example, the United Kingdom of Great Britain and Northern Ireland (GB) [10] and Japan [11] both appointing a Minister for Loneliness in 2018 (GB) and 2021 (Japan) respectively. In a joint press statement, both an EU Commissioner and the Japanese Loneliness Minister agreed that “loneliness and social isolation pose crucial challenges to the cohesion, economy and mental and physical health in 21st century societies across the world” [12]. In November 2023, the World Health Organization highlighted the importance of social connection, recognising the significant and often underestimated impact of loneliness and isolation on our health and well-being. This recognition led to the launch of its Commission on Social Connection (2024–2026), which aims to address this issue as a public health concern [13]. However, little is known about the extent that loneliness is currently included in national strategies and policies across the world.

Loneliness is often defined in psychological terms as an unpleasant feeling that people experience when they perceive their social relationships to be qualitatively or quantitatively inadequate [14]. The quality, rather than the quantity, of social relationships plays a greater role in loneliness [15]. While temporary loneliness is a natural human experience, chronic loneliness has serious negative consequences for health and life expectancy. There are three main types of loneliness: intimate (also known as emotional) loneliness, relational (also known as social) loneliness and collective loneliness, first identified by McWhirter (1990) [16], and empirically validated by Hawkley et al. (2005) [17] and Panayiotou et al. (2023) [18]. Loneliness is distinct from social isolation, which Nicholson Jr. (2009) [19] defines as “a state in which the individual lacks a sense of belonging socially, lacks engagement with others, has a minimal number of social contacts, and they are deficient in fulfilling and quality relationships” (p. 1346). This does not mean that socially isolated individuals necessarily feel lonely and vice versa.

There are different scales to measure loneliness and social isolation. The most commonly used instruments for measuring loneliness are the indirect measures from De Jong Gierveld Loneliness Scale [20] and the full UCLA Loneliness Scale [21], as well as the direct measure from the UK Office for National Statistics [22]. However, what these definitions fail to measure is the “intensity, frequency and duration of loneliness. Loneliness can be acute (i.e., transient) or chronic (i.e., enduring), and it can be mild to severe in its intensity” [23, p.2]. There are also a variety of scales to measure social isolation, but there is no consensus on which should be used [24]. Some common scales include the Lubben Social Network Scale [25], the Cudjoe social isolation typology [26] or a social isolation index used by Shankar et al. [27].

Our study contributes to existing literature by presenting an overview of current governmental documents that address loneliness and social isolation. Our intention is that the scoping review would be used by federal agencies or local communities who want to develop their own strategies to address loneliness and social isolation, or by researchers to gain an overview of the policy landscape.

### Study aims

The aim of this study was to characterise the policy landscape relevant to tackling loneliness and social isolation across the UN European country groups to identify commonalities and differences between national approaches to loneliness. A secondary aim was to provide actionable recommendations including their implications based on the scoping review for the consideration of policy makers to help promote the rapid and widescale adoption and diffusion of sustainable, scalable and evidence-based interventions to manage loneliness.

## Methods

We conducted a scoping review based on Mak and Thomas’ recommendations (2022) [28] to identify (i) how loneliness and social isolation are defined, (ii) the common characteristics between loneliness policies across countries, (iii) which population groups were targeted, and (iv) whether there was an identifiable commitment to action and funding. We contextualised findings using five domains (geographic, social, health, economic, political) that all affect or are affected by experiences of loneliness and social isolation. We have taken every step to make the scoping review as clear and reproducible as possible, following the PRISMA-ScR guidelines [29] [see file: Supplementary Material_PRISMA-ScR-Checklist].

### Eligibility criteria

A multi-method review approach inspired by Schnable et al. (2021) [30], including a qualitative policy analysis, was used to identify and describe the characteristics of a collection of national-level government documents with reference to loneliness and social isolation. As national policy documents and commissioned governmental strategies and action plans are not available on a central database, a systematic review was not feasible.

We retrieved and reviewed policy documents that address loneliness or social isolation from a total of 52 countries from the UN European Country Groups: Albania (AL), Andorra (AD), Armenia (AM), Australia (AU), Austria (AT), Azerbaijan (AZ), Belarus (BY), Belgium (BE), Bosnia and Herzegovina (BA), Bulgaria (BG), Canada (CA), Croatia (HR), Czechia (CZ), Denmark (DK), Estonia (EE), Finland (FI), France (FR), Georgia (GE), Germany (DE), Greece (GR), Hungary (HU), Iceland (IS), Ireland (IE), Israel (IL), Italy (IT), Latvia (LV), Liechtenstein (LI), Lithuania (LT), Luxembourg (LU), Malta (MT), Monaco (MC), Montenegro (ME), Netherlands (NL), New Zealand (NZ), North Macedonia (MK), Norway (NO), Poland (PL), Portugal (PT), Republic of Moldova (MD), Romania (RO), Russian Federation (RU), San Marino (SM), Serbia (RS), Slovakia (SK), Slovenia (SI), Spain (ES), Sweden (SE), Switzerland (CH), Türkiye (TR), Ukraine (UA), United Kingdom of Great Britain and Northern Ireland (GB), and United States of America (US). We chose this geographic focus of Europe because the European Union was the first supranational union of states to put loneliness on its agenda with a policy brief published in 2018 [31]. To ensure comprehensive coverage of European nations, we chose the UN European country groups, recognising that they include some members beyond the continent’s geographical borders.

Articles including policies, reports, strategies and policy briefs were included in the analysis if they were (i) from the two UN country groups under study, (ii) officially published or commissioned by a national government, (iii) publicly available, (iv) published between 1 January 2003 and 1 July 2023, (v) related directly to loneliness and social isolation or indirectly by using other language such as social connection, (vi) published in any language.

### Information sources

The main information sources were governmental websites of relevant ministries and departments of the 52 selected countries. Additionally, we used the Google search engine for all publicly available national policies related to loneliness and social isolation.

### Search

We conducted desktop research using the key terms “loneliness” and “social isolation” for all publicly available national policies, including a review of government websites to generate an asset map of key policy documents and white papers from each country. Online searches were conducted between 1st February 2023 and 1st July 2023.

Internet searches, using the Google search engine, included the following keywords: [(“loneliness” OR “social isolation” OR “social connection”)] and [(“policy” OR “strategy” OR “actions” OR “reports”) and “Country”]. If this did not yield any results for a specific country, we searched for the government website of that country using primary (loneliness and social isolation) and secondary (strategy/policy) terms to determine if governments published documents on loneliness and social isolation. The Google website translator was used to navigate non-English governmental websites.

### Selection of sources of evidence

The documents were not limited to policies, but also included national strategies, technical reports, brochures and webpages published by government agencies, studies commissioned by a government agency, governmental press releases, and parliamentary enquiries from politicians to federal ministers or councillor regarding data on loneliness in their respective countries. If multiple strategies/policies from the same government were found, the most recently published one was included. We focused on national level documents only (excluding any regional strategies).

Where documents retrieved were not in English, they were translated into English using a paid (subscription) version of DeepL Pro, a powerful and sophisticated online translator. For reasons of pragmatism, no attempt was made to quality assure the translation with native speakers.

We excluded 40 documents after a first round of reviews where there was no disagreement between the researchers. For 20 documents there was no consensus, so a third researcher reviewed the documents. After reviewing each document, consensus was reached to exclude 16 of the 20 documents. Documents were excluded for the following reasons: (i) loneliness and social isolation were only mentioned in passing and did not elaborated on the issue of loneliness, or loneliness was not part of a proposed intervention, (ii) highlighted or acknowledged loneliness as a problem but we could not identify any detail or strategies or commitments on how to address it, (iii) short news piece or press releases that did not specifically touch on loneliness or social isolation, (iv) documented queries raised by political representatives addressed to parliament, (v) research articles not commissioned by the government, (vi) local focus, not national, (vii) NGO reports not commissioned by a government and (viii) older versions of included documents.

### Data charting process

The principal investigator (NG) developed a coding matrix using Excel based on the study objectives and considerations from Braun and Clarke (2006) [32]. This matrix was first tested on the British documents (NG, DK, MLEA), as we knew these to be extensively detailed. In an iterative process this matrix was reviewed and adapted after testing it on a random selection of five sources of evidence (NG, DK, MLEA, PQ). After a final round of reviewing and adapting, all authors agreed by consensus that they have captured all desired variables needed to address the study objectives. Each policy document was coded independently by at least two investigators (NG, DK, MLEA) to minimise human error in information extraction.

### Data items

The configuration matrix was completed for all sources of evidence containing information on: (i) document overview (title, publisher, year of publication, original language of publication), (ii) recommended measurement tool for loneliness, (iii) definitions for loneliness, social isolation and other language around social connection, (iv) target group of policy, (v) proposed or suggested actions by government (raising awareness, funding pledge, call for a development of a loneliness measure, proposed interventions or actions, type of evidence cited, commitment to work with specific charities), and (vi) five key domains (geographic, social, health, economic, political) that affect or are affected by experiences of loneliness and social isolation. We also coded whether the documents referred to five domains (geographic, social, health, economic, political) that have been shown to affect or are affected by experiences of loneliness and social isolation.

### Synthesis of results

The data of the configuration matrix were consolidated and are presented as Table 1, Supplementary Table A [see file: Supplementary Material_Table  A], and within the text where a presentation in table format was not deemed useful (for data items 3–5 as detailed above). We used the document analysis as proposed by [33] to analyse all the included documents. This approach is based on an iterative processes of qualitative content analysis [34], with a specific thematic analysis [32]. The configuration matrix captured all extracted data from which the authors (NG, DK, MLEA) could identify emerging sub-themes within these broad pre-defined domains of loneliness (geographic, social, health, economic and political domain) using thematic analysis [32]. To create recommendations, two authors (NG, PQ) reviewed the extracted data, with the team revisiting the sources of evidence where needed.

**Table 1:** Overview of included documents (*n* = 23 documents, 14 countries)

Country [abbr.] (source)	Document Name	Document Type	Government Department	Year	Identifiable funding pledge?	Intervention proposed?	Target group
Albania [AL] [35]	The National Action Plan on Aging	Action Plan	Ministry of Health & Social Protection	2019	Yes	Yes	Older adults (aged 65+)
Australia [AU] [36, 37]	Social Isolation & Loneliness	Government website	Australian Institute of Health and Welfare	2021	Yes	Yes	General population
	A National Strategy to Address Loneliness and Social Isolation	Strategy	Hosted at The Treasury of the Australian Government. Strategy produced by Ending Loneliness Together in partnership with R U OK? and the Australian Psychological Society	2021	Yes (call for funding)	Yes	Government
	Understanding and Defining Loneliness & Social Isolation	Resource sheet	Australian Institute of Family Studies, Dept of Social Services	2022	No	Yes	General population, with specific vulnerable groups
Austria [AT] [38]	Overcoming Loneliness	Government website	Federal Ministry of Social Affairs, Health, Care and Consumer Protection	2023	No	Yes	General population
Canada [CA] [39, 40]	Report on the Social Isolation of Seniors	Technical Report	National Seniors Council	2014	No	No	Older adults (aged 65+)
	A Profile of Social Isolation in Canada	Technical Report	Hosted at Ministry of Health. Report produced by university researchers	2006	No	No	Older adults (aged 65+)
Czechia [CZ] [41]	Social Inclusion Strategy 2021–2030	Strategy	Ministry of Labour & Social Affairs	2019	No	Yes	General population
Denmark [DK] [42, 43]	The Danish National Strategy Against Loneliness	Strategy	2 NGOs: Ældre Sagen (= The Elderly Association), General Red Cross on behalf of the national government	2023	Yes (call for funding)	No	General population
	Danish Action Plan Against Loneliness	Action Plan	2 NGOs: Ældre Sagen (= The Elderly Association), General Red Cross on behalf of the national government	2023	Yes	Yes	General population
Germany [DE] [44]	Loneliness - recognising, evaluating & resolutely confronting it	Technical Report	Committee for Family Affairs, Senior Citizens, Women and Youth	2021	No	Yes	General population
Ireland [IE] [45]	Stronger together- The HSE Mental Health Promotion plan 2022–2027	Strategy	Health Service Executive Ireland Mental Health and Wellbeing Programme + National working Group	2022	No	Yes	General population, with specific vulnerable groups
Italy [IT] [46]	Policies for active ageing in Italy: what are the possible objectives?	Policy	Family Department	2022	No (only mentions need for funding)	Yes	Older adults (aged 65+)
Malta [MT] [47]	National Strategic Policy for Active Ageing 2023–2030	Policy	Ministry for Senior Citizens and Active Ageing	2022	No	Yes	Older adults (aged 65+)
Netherlands [NL] [48]	One against loneliness. Action programme 2022–2025.	Action Plan	Ministry of Health, Welfare and Sport	2022	Yes	Yes	General population, with specific vulnerable groups
Switzerland [CH] [49, 50]	Social resources as health protection: Mode of action and dissemination in the Swiss population and in Europe	Technical Report	Swiss Health Observatory	2014	No	No	General population, with specific vulnerable groups
	Social resources: Promotion of social resources as an important contribution to mental health, health and a high quality of life	Technical Report	Health Promotion Switzerland	2020	No	No	General population, with specific vulnerable groups
United Kingdom [GB] [51–53]	Emerging Together: The Tackling Loneliness Network Action Plan	Action Plan	Department for Digital, Culture, Media and Sport	2021	Yes	Yes	General population, with specific vulnerable groups
	A connected society - A strategy for tackling loneliness	Strategy	Department for Digital, Culture, Media and Sport	2018	Yes	Yes	General population
	Wellbeing in Northern Ireland	Technical Report	NISRA, Northern Ireland Statistics and Research Agency	2022	No	No	people aged 16 and over in Northern Ireland
United States of America [US] [54–56]	Social Isolation and Loneliness in Older Adults: Opportunities for the Health Care System	Study	The National Academies of Science, Engineering, Medicine	2020	No	Yes	Older adults (aged 65+)
	Addressing Social Isolation to Improve the Health of Older Adults: A Rapid Review	Study	Agency for Healthcare Research and Quality (US Department of Health and Human Services)	2019	No	Yes	Older adults (aged 65+)
	Our Epidemic of Loneliness and Isolation. The U.S. Surgeon General’s Advisory on the Healing Effects of Social Connection and Community.	Advisory	U.S. Public Health Service	2023	No	Yes (broadly phrased)	General population

## Results

### Selection of sources of evidence

**Fig. 1:**
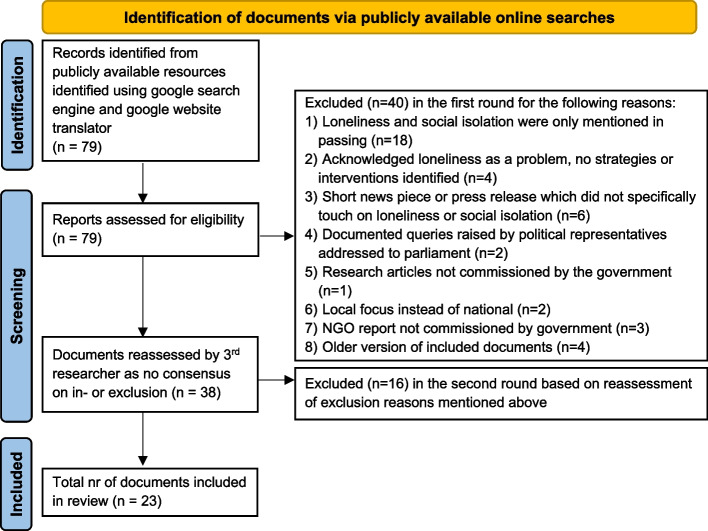
PRISMA flow chart based on [24]

Our scoping review identified 79 sources of evidence that discussed loneliness and social isolation from across 32 countries in both UN European country groups. We excluded a total of 56 documents after two review rounds for reasons shown in the PRISMA flowchart Fig. 1. This yielded a subset of 23 documents that were included in our final analysis.

### Wider awareness of loneliness and social isolation in our study area

**Fig. 2:**
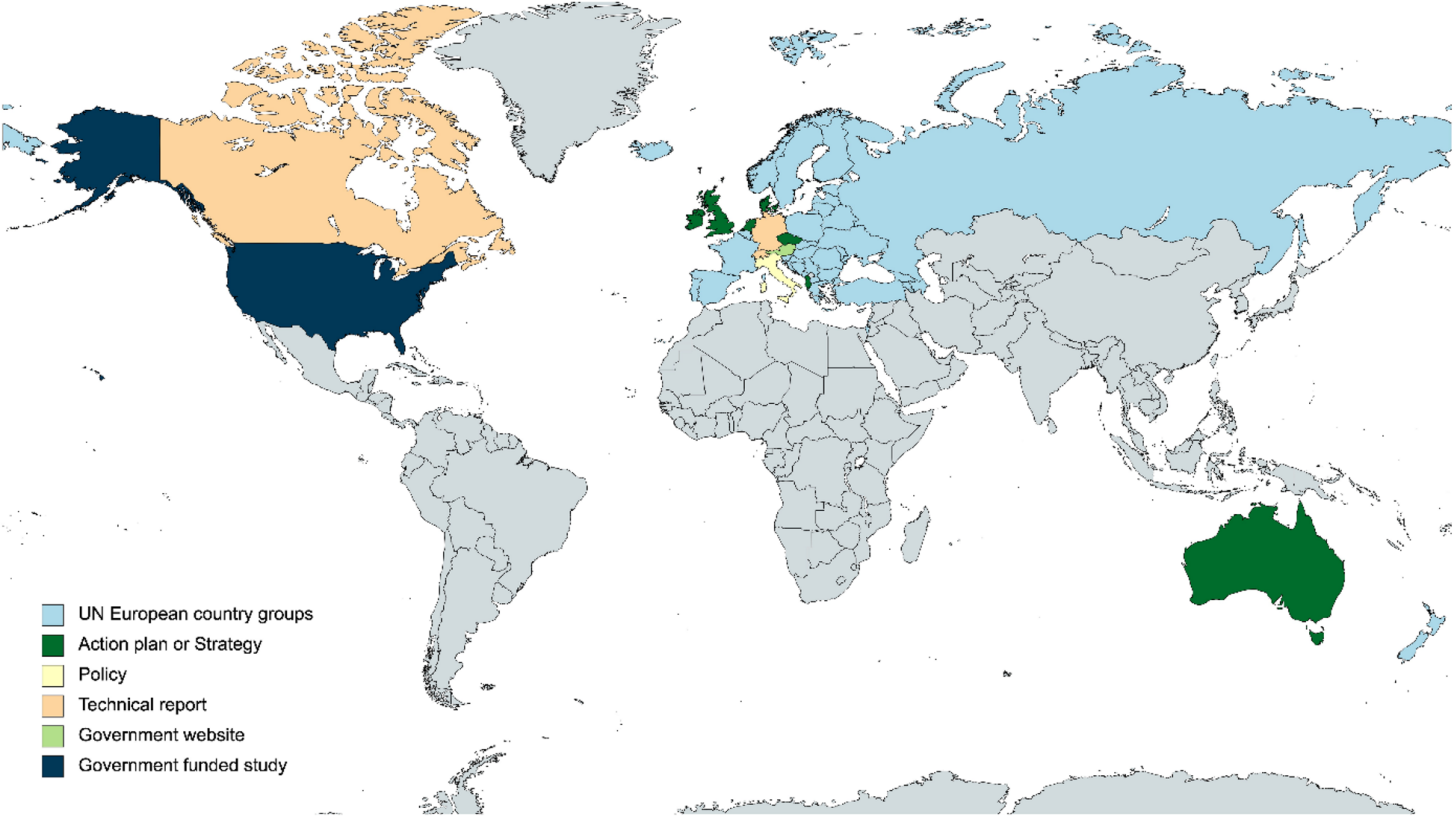
Current state of the loneliness policy landscape across the study area. Map created with [28]

Here, we delve into the sources of evidence that were excluded from our study, but which are nonetheless noteworthy because they illustrate the momentum of the international conversations around loneliness. In some countries (AT, CH), we found parliamentary enquiries asking about data on loneliness in their respective countries, and whether there were any strategies in place to alleviate loneliness. DE does not have a loneliness strategy, but the governmental Committee for Family Affairs, Senior Citizens, Women and Youth has partially funded the organisation (the Competence Network on Loneliness (KNE)) which looks at the causes and consequences of loneliness and promotes the development and exchange of possible prevention and intervention measures in DE. NZ is a good example where there was no specific policy, despite there being great public awareness. They have an established nationwide trust called “Loneliness New Zealand Charitable Trust”. While some countries had excellent resources targeted at policy makers (e.g. CA), they have not yet been translated into a nationwide policy to address loneliness and social isolation. In countries where there was no national strategy, some cities have designed their own regional strategies or organisations, e.g. Barcelona [57], Helsinki [58], or Vancouver [59]. A map highlighting the loneliness policy development landscape across 52 countries of the UN European Country Groups is shown in Fig. 2.

It is important to note that for many countries in the study area we could not identify any resources that met the inclusion criteria. It is difficult to assess why loneliness and social isolation are not on the policy agenda of more national governments. Connel and t’ Hart [60] have developed a typology of policy inaction. Three of the five types may apply to our context: Type I: Calculated inaction. Governments may make a strategic decision not to act, or not to act now, because they believe that the costs of action outweigh the perceived benefits, or because they want to see a stronger evidence base on an issue. Type II: Ideological inaction. Government inaction as a product of ideology, where governments rely on non-governmental and not-for-profit organisations to address the issue of loneliness. The strong third or social-economy sector in the European Union [61], which includes more volunteers than paid employees, could give the impression that loneliness and social isolation can be managed without government policies. Type IV: Reluctant inaction. Governments do not act because they perceive an absolute or relative lack of resources to fund loneliness and social isolation policies. This may be the case for the less economically strong countries in our study area that do not have policies in place.

### Characteristics of sources of evidence

Table 1 gives an overview of the 23 documents that we included in our analysis. Half the documents were published after 2020. Seven documents had to be translated into English. Certain countries released documents in conjunction with one another. For instance, Denmark published a National Strategy and an Action Plan simultaneously in 2023 that were complementary. Similarly, GB’s 2021 Action Plan builds on the GB Loneliness Strategy published in 2018.

### Results of individual sources of evidence

For each of the included sources of evidence, we extracted information with our configuration matrix presented in the section Data items. We believe that presenting the results this way will better suit our study objectives, i.e., to highlight common and unique themes.

### Target group of policies

Eight documents (from AL, CA, IT, MT, US) were targeted specifically at the older adult population, often classified as age 65 + years. Definitions, causes and proposed interventions for loneliness and social isolation in those documents were contextualised within the framework of old age. The other documents addressed the general population, often highlighting that there are specific groups that are more vulnerable to becoming lonely or socially isolated. Five of the documents identified target groups at increased risk of loneliness (AU, IE, CH, GB, DK). For instance, children (IE), young adults ages 18–25 years (AU, DK, IE, GB), older adults ages 65 + years (AU, CH, IE, GB), people with disabilities & special needs (AU, DK), people suffering from mental illness (CH), those with long-term illness (GB), migrants and refugees (AU, CH, GB), lower income households (AU), and people living alone (AU, CH), people with lower levels of schooling (CH), single parents (CH), young single men (CH), care leaver (GB), victims of domestic violence (US), LGBTQ + individuals (US) and minorities (DK, US).

### Defining loneliness and social isolation

Of the 23 documents included in the review, 11 documents from seven countries (AU, AT, CA, DE, NL, GB, US) provided specific definitions of loneliness and social isolation. Those definitions were based on academic sources, explicitly referenced and cited, except for AT which based their definition on general “experts” rather than a specific source. Peplau and Perlman (1982)’s widely used framework is drawn upon in multiple documents, and some countries (AU, DE, NL, GB) go further in their definitions to distinguish between different types of loneliness, (e.g., social, emotional, and existential loneliness in the NL document).

The 11 documents that used a specific definition of loneliness used the Peplau and Perlman (1982) definition that highlights differences between loneliness and social isolation. Documents noted social isolation as an objective lack of social relationships, while loneliness is considered to be the subjective feelings as a result of that social isolation.

Across all the documents included in our review, both with and without specific definitions of loneliness, other language used around social connection can be classified as follows:

1. Inclusion in wider society, which includes the terms social inclusion (CZ, DK, IE, MT), social integration (CA) and social participation (DE, NL, CH).
2. Connecting with others, which includes the terms social networks (CA, DK, DE), social support (CH, US), social connection (AU, US), and social contacts (AT, DE, NL, GB).
3. Existing resources, which includes the terms social resources (CH), social capital (CH, CA), and social skills (CA, NL).
4. Covering a deficit, which includes the terms social exclusion (AL, CZ, CA), social vulnerability (IT, CA) and social recovery (AU).
5. Relationship between loneliness and mental health, which includes the term social wellbeing (GB), and discussions of social prescribing (GB) and the contribution of loneliness to poor mental health (IE).
6. Mental health, which includes the term social wellbeing (GB), and discussions of social prescribing (GB) and the contribution of loneliness to poor mental health (IE).

### Funding pledges

Despite the governmental strategies and action plans to reduce loneliness and social isolation, we found little evidence of a commitment to funding. We identified concrete funding pledges or already provided funding for AL (0.75 m USD for 5 years), DK (145 m USD for 2014–2025), GB (24.8 m USD in 2018; 44.5 m USD in 2020), and NL (10.7 m USD per year for 2022–2025; 5.5 m USD 2018–2022) governments. DK provided a detailed overview of initiatives that can be achieved within the already approved budget, initiatives that could be delivered within existing financial frameworks and over 80 initiatives that should be advanced but required additional funding. The Australian government has not yet made a funding pledge but has received a specific budget and initiative proposal for funding from an alliance of three different national organisations. Other government strategies either stated that different ministries are to ensure the necessary financial and human resources for initiatives that fall under their respective jurisdiction (MT) or did not specify funding pledges, merely stating that adequate funding needs to be identified (IT). We identified that some governments (DE, SE) are (partially) funding research on loneliness to gather scientific evidence to help them build their own policy.

### Interventions and partnerships

Strategies, policies and action plans proposed a variety of interventions, while technical reports focused on reviewing existing evidence. We have provided many intervention examples across various domains in the policy landscape analysis section below. Of those countries and documents included in our analysis, only AU and GB have committed to work with specific charities, organisations or initiatives to address loneliness and social isolation. Other governments (CA, IE, IT, MT) stated their intention to work with NGOs and local services, but did not mention any specific organisations.

### Development of a loneliness and social isolation measure

None of the documents called for the development of new tools to measure loneliness or social isolation. US, DK and GB reviewed existing measures of loneliness for use in possible interventions and strategies. Notably, GB described its own use of a consistent and direct measure of loneliness, developed by the Office of National Statistics (ONS) in 2018. The Direct Measure of Loneliness is a single item measure developed by the ONS that should be used in conjunction with three questions from the University of California Los Angeles (UCLA) Loneliness Scale. A US documents considered multiple ways in which loneliness and social isolation should be measured in research and recommended the appropriate choice of measures in targeted interventions and in major health strategies. The US did not call for the creation of a new measure, but rather recommended the use of existing validated tools tailored to the purpose of proposed interventions. DK’s national strategy considered the applicability of adult measures to adolescents and children.

### Policy landscape analysis

This section highlights the wider policy context of the loneliness debate. All 14 countries that have published documents on loneliness are aware that loneliness touches many different dimensions (geographical, health, social, economic, and political; see Table 2 for a brief overview). In 91% (*n* = 21) of the analysed documents, the social and health dimension was most prominent, highlighting the impact of loneliness on various aspects of people’s lives and across age groups, as well as the health implications. However, not all dimensions were addressed with the same level of detail. An extensive overview of the different dimensions touched upon in every document can be found in the Supplementary Table A. For each of the five dimensions, we have identified themes that recur across the documents. We have also added some intervention examples to show how loneliness could be addressed in this dimension from a policy perspective.

**Table 2:** Prevalence of geographic, social, health, economic and political dimensions in loneliness and social isolation documents

Document title (Country abbreviation)	Geo-graphic	Social	Health	Economic	Political
The National Action Plan 2020–2024 (AL)	Yes	Yes	Yes	Yes	Yes
Social Isolation and Loneliness (AU)	Yes	Yes	Yes	Yes	Yes
A National Strategy to Address Loneliness (AU)	--	Yes	Yes	Yes	--
Understanding and Defining Loneliness and Social Isolation (AU)	--	--	--	--	--
Overcoming Loneliness (AT)	Yes	Yes	Yes	Yes	--
Report on the Social Isolation of Seniors (CA)	Yes	Yes	Yes	Yes	--
A profile of Social Isolation in Canada (CA)	Yes	Yes	Yes	Yes	--
Social Inclusion Strategy 2021–2030 (CZ)	Yes	Yes	Yes	Yes	--
The Danish National Strategy Against Loneliness (DK)	Yes	Yes	Yes	Yes	Yes
The Danish Action Plan Against Loneliness (DK)	Yes	Yes	Yes	Yes	Yes
Loneliness- recognising, evaluating and resolutely confronting it (DE)	Yes	Yes	Yes	Yes	Yes
Stronger Together- The HSE Mental Health Promotion Plan 2022–2027 (IE)	--	Yes	Yes	Yes	--
Policies for Active Ageing in Italy: What are the Possible Objectives? (IT)	Yes	Yes	Yes	Yes	--
National Strategic Policy for Active Ageing (MT)	Yes	Yes	Yes	--	--
One against loneliness. Action Programme 2022–2025. (NL)	Yes	Yes	Yes	Yes	Yes
Social Resources as Health Protection: Mode of Action and Dissemination in the Swiss Population and in Europe (CH)	--	Yes	Yes	Yes	--
Social Resources-Promotion of social resources as an important contribution to mental health. Health and a high quality of life (CH)	--	Yes	Yes	Yes	--
Emerging Together: The Tackling Loneliness Network Action Plan (GB)	Yes	Yes	Yes	--	--
A Connected Society. A strategy for tackling loneliness (GB)	Yes	Yes	Yes	Yes	--
Wellbeing in North Ireland 2021/22 (GB)	Yes	Yes	Yes	--	--
Social Isolation and Loneliness in Older Adults: Opportunities for the Health Care System 2020 (US)	Yes	Yes	Yes	--	--
Addressing Social Isolation to Improve the Health of Older Adults: A Rapid Review (US)	--	--	--	--	--
Our Epidemic of Loneliness and Isolation: The U.S. Surgeon General’s Advisory on the Healing Effects of Social Connection and Community (US)	Yes	Yes	Yes	Yes	Yes
**TOTAL n (%)**	**17 (74%)**	**21 (91%)**	**21 (91%)**	**17 (74%)**	**7 (30%)**

### Geographic dimension

Most documents (74%, *n* = 17) touched on various geographic dimensions that influence or are influenced by loneliness. Four governments observed geographical variation in loneliness prevalence within their country (AU, CA, DE, GB). Only one document suggested reforming the digital environment (US). Within the geographic dimension the following themes were most often mentioned as being influential regarding loneliness and social isolation in the context of geography: (i) place or residence and housing, (ii) public transport, (iii) community services, and (iv) urban planning.

#### Place of residence and housing

Four governments (AU, CA, DE, GB) reported that the place of residence (urban or rural) significantly influences loneliness. Loneliness levels were also considered to vary due to population changes (AT, DE) but acknowledged that regional distribution was complex and cannot be solely attributed to urban-rural differences. Relocating to a new place was also reported to lead to feelings of being disconnected from familiar social networks and support systems. Additionally, insufficient affordable and suitable housing contributed to social isolation. Living conditions were mostly mentioned in connection with older adults where the effect of the type of housing was mentioned to affect social interactions and feelings of loneliness (CA, DK). Intervention examples to manage loneliness as a result of a change in residence, or loss of housing include working within local municipal authorities’ strategies on housing policies and reform plans (IT, DK, NL), creating models of apartments that foster community life (AL, DK), creating flexible housing solutions to support life transitions, e.g. homes that can be adjusted in size or adapted to changing needs (DK).

#### Public transport

The impact of public transport, especially access and affordability, was mentioned as a key issue for social integration, especially for older people (AL, CA). The place of residence (especially if rural) was recognised as a barrier to public transport use. Intervention examples that were put in place to address this issue include an increase of public transport access for the poorer older adults by subsidising the costs locally (AL, DK), and further strengthening accessible transport for communities in residential areas specifically (DK, GB).

#### Community services

Limited awareness of or access to community services contributed to loneliness. Financial support and grants for rural projects are needed to promote social inclusion. GB, DK and NL documents highlight the importance of the central government working together with local authorities, as the latter play a key role in actively supporting local transport, voluntary groups and initiatives that promote social cohesion and reduce isolation. Intervention examples included subsidies for community work to promote social inclusion specifically in rural areas (CZ), expanding the services in and of community centres (AL), and promoting the use of tailored community-based services (US).

#### Urban planning

There was general awareness that the physical environment can pose challenges to social participation, especially for the more vulnerable groups, e.g. older adults (CA), in terms of access to public toilets or walkability. Intervention examples included cultivating a sense of belonging that should be considered by urban planners (CA, IT), ensuring proximity to public services (IT), access to public toilets (CA), establishment of healthy and active movement paths (IT) aimed at encouraging walking groups (IT, CH), maximising the use of underutilised community spaces (GB), and use of participatory design in the development of child-friendly neighbourhoods in local environments (CH).

#### Social dimension

Most documents (90.9%, *n* = 20) highlighted a range of interrelated social factors associated with loneliness; the social determinants covered various aspects of people’s lives that shape experiences of loneliness across age groups. Throughout these documents were notes on groups more vulnerable to loneliness as well as everyday life transitions and triggers. Some risk factors for loneliness such as lacking contact with family and friends, the negative impact of unemployment, and inadequate income support were also prominently highlighted.

#### Groups vulnerable to loneliness

Many governments identified groups more vulnerable to loneliness and social isolation, in line with research findings (AL, CA, IT, MT, NL, CH, GB, US). The following groups were identified as more vulnerable to becoming lonely or socially isolated: single parents, widows, newly retirees, single households, those living in changing family structures, immigrants with language barriers or low socioeconomic status, individuals dealing with addiction, those from the LGBTIQ + community, young adults (around 18 to 29), older adults (above 80), individuals that experience bullying or harassment, and individuals with criminal records. The importance of cultivating inclusive communities and establishing safe spaces for individuals, particularly for groups like migrants, single parents, and older adults was emphasized. Interventions were often tailored to specific groups. For example, community-led interventions targeted older adults who were homebound or in residential long-term care (MT). Others strengthened the resources of older people caring for relatives (CH), invested in a Carers Action Plan (GB), levelled up the volunteering infrastructure through collaboration of the voluntary sector and the government especially for those out-of-work (GB), developed social prescribing pilots and peer support groups (GB, US), facilitated befriending and socializing (AU), and linked vulnerable groups of people in the form of self-help and enabled them to help each other (CH). Here are some examples of targeted interventions for specific groups:

- Women: language classes for women who do not speak the local language with crèche facilities alongside the classes (GB), Mitigate the risks of lifelong gender inequalities that result in female old-age poverty and gender pension gaps by ensuring adequate levels of income security for older women (MT).
- Men: increase offers for older (single) men such as Men’s Meeting Places or Men’s Communities (DK), active aging centres to mitigate against the tendency of older men to experience difficulties in seeking help and talking about loneliness (MT).
- Young people: Strengthen detection of loneliness in day care, primary schools and educational institutions (DK), provide education courses as a source of mitigating loneliness among children (DK), create more binding communities for young people without education and jobs (DK).
- Older adults and low-income households: offer free local cultural and leisure activities (CH), increase public transport access (AL), guaranteeing the living minimum and gradual improvement of lowest pensions (AL), activation of computer literacy paths (IT).

### Everyday life

The impact of events like the pandemic on individuals and communities was noted, with reference to mental well-being and social interactions, including potential changes in post-pandemic work patterns that might limit personal engagement. The absence of support or opportunities within society, communities, and workplaces is discussed as hindering social integration and fosters loneliness. The role of technology and social media as both a potential mitigating and exacerbating factor was recognized. Intervention examples include enhancement of popular traditions by developing new forms of technologically-oriented interactions, while still including cultural heritage (IT), expansion of existing community interventions (MT) including specific funding allocated to national, local, and community levels (AU), development of national and community awareness or anti-stigma campaigns (AU, CA, DK, DE, IE, NL, GB, US), and awareness spreading specifically towards politicians, administrations, managers, health care providers and others who work on loneliness (DK, US).

### Health dimension

The health dimension of loneliness was very prominent in most documents (91%, *n* = 21), often noting that socially isolated individuals faced an increased risk of engaging in negative health behaviours. The evidence of interconnection between chronic illnesses, mental health and social isolation was also highlighted. Overlapping with recommendations identified in the social domain, the need for policy development to prioritize social function among older individuals, aiming to enhance their overall health and well-being, was mentioned by (AT, DE, IE).

Institutional intervention examples included the development of an integrated health and social system on a community basis (AL, DK), national training for health practitioners and community care services to systematically identify, monitor and direct people experiencing loneliness (AU, DK, MT, US), linking healthcare practitioners with researchers to further evaluate and use loneliness assessment tools in clinal settings (US), and the inclusion of loneliness and social isolation in electronic health records (AU, US).

#### Physical health

Documents noted the evidence that individuals with higher levels of chronic diseases, geriatric syndromes, reduced mobility, chronic pain, frailty, hearing and sight impairment, urinary incontinence, or other health issues necessitating long-term care were more susceptible to loneliness. Governments acknowledged these links, often targeting interventions to support disabled people. Intervention examples included the provision of sensory impairment guides for those whose social lives are impacted by a change in their senses due to accidents or disabilities (GB), strengthening bridge-building for civil society and other actors was recommended in the context of in-system transitions and among high-risk groups (DK), the establishment of mobility centres to help people stay mobile or provide information on alternative modes of transport (GB), increased focus on digital inclusion of older and disabled to reduce loneliness as they face reduced mobility (GB), and the advancement of physical activity interventions, especially promising for improving the health outcomes of older adults (US).

### Mental health

The policy documents showed empirical evidence that individuals experiencing depression, mental health problems and addiction were at risk of social exclusion. Depression and anxiety are specifically mentioned as significant factors in the context of loneliness; the consequences of loneliness are also discussed, with reference to the increased risk of depression, suicide, anxiety disorders, dementia, and reduced cognitive abilities. Intervention examples included the introduction of community care for people with mental health problems (CZ), while others focused attention on cognitive behavioural therapy, interpersonal psychotherapy and mindfulness (US). The reduction of addictive substances in populations at risk of social exclusion was targeted (CZ); mental health literacy programs were also discussed (DE, IE), specifically in reference to school education initiatives such as social emotional learning programs for use in preschool, school, and youth settings (IE); mental health literacy campaigns were also highlighted (DE, IE).

### Economic dimension

Economic factors relating to loneliness were also addressed most documents (74%, *n* = 17). In line with research evidence, documents noted that unemployment, receiving income support, and dissatisfaction with financial situation contribute to loneliness. The need for allocating more resources to combat poverty and address the loneliness experienced by older individuals was emphasised, with reference to the fact that it plays a crucial role in enhancing their overall well-being and quality of life. The following themes were prominent within this dimension.

#### Income

Economic poverty stemming from insufficient income was identified as a key concern for the older adult population. Notably, social exclusion and family poverty were found to be directly linked, posing a risk to children as well. One document (AU) noted that men ages 25–44 years with high incomes and women of all ages with low incomes have been to be more susceptible to loneliness, revealing a discrepancy based on gender. The economic burden of loneliness extended to health service utilization costs, especially for mental health services. Intervention examples included allocating more resources to combat poverty and address the loneliness of older people specifically (IT), guaranteeing dignified living conditions through the adoption of the minimum pension and the gradual improvement of the lowest pensions by offering sustainable support for the poorer elderly was also suggested within the economic domain (AL), early support interventions for children from disadvantaged families, including support for their parents (CH), and more widely to reduce risk of social exclusion due to over-indebtedness (CZ).

### Unemployment

Lack of affordable and suitable housing and care options was noted as being linked to social isolation. Loneliness and lack of social support could lead to reduced community participation, hindering employment prospects and workplace progress. This can result in reduced productivity, lower job satisfaction, increased absenteeism, and longer recovery times due to stress and health issues, which in turn negatively affects the economy. Intervention examples included facilitation of the integration of vulnerable individuals into the workforce (CZ, DK), prevention of loneliness among the unemployed through volunteerism and community initiatives (GB, DK), focus on ensuring a smooth transition from work to retirement (DK), working in collaboration with job centres (GB), and creating a cultural shift in work environments for employees at risk of social exclusion (CZ).

### Political dimension

Political factors pertaining to loneliness and social isolation were only identified in few documents (30%, *n* = 7), indicating less governmental awareness of the political implications of loneliness. Instances of elderly individuals being denied many rights were observed to be associated with loneliness (AL). Additionally, the effects of COVID-19 lockdown policies were connected to the loneliness because of social isolation. DE mentioned the political relevance of loneliness as it correlates with decreased political engagement of individuals. Thus, it was stated that implementing political measures at the federal level is imperative to effectively foster a more socially connected society (DE). One of the documents mentioned the need for the government to establish a comprehensive national strategy targeting loneliness, accompanied by the allocation of sufficient funding, with active engagement from regions and municipalities, especially when it comes to implementation (DK). Furthermore, the same document underscored the contribution of various other key stakeholders, including research institutions, foundations, employers, and civil society, in combating loneliness (DK). Multiple countries acknowledged the relevance of working across government bodies and levels in combatting loneliness (AU, DE, NL, GB). One document highlighted the need for a “connection-in-all-Policies” [62, p.49] approach as social connection, an antidote to loneliness and social isolation, is relevant in all sectors (US).

## Discussion

To our knowledge, this is the first study to characterise the loneliness policy landscape across the UN European country groups (52 countries). The scoping review provided comprehensive coverage of how countries address loneliness and social isolation on a national level, allowing for a much clearer understanding of the diversity in country-level strategies and better coordination across countries in tackling loneliness. This is particularly important because loneliness and social isolation have been increasingly identified as a public health concern [63, 64]. The findings of this review can be used by a wide range of stakeholders including federal agencies and local community groups who want to develop their own strategies to address loneliness and social isolation, or by researchers to gain an overview of the policy landscape.

### Summary of principal findings

While not all governments (14 of 52 countries; 27%) had official documents that addressed loneliness, the vast number of documents we identified (79 documents) highlight the growing momentum in the loneliness discourse in the study area. The inclusion of research findings in the vision and strategy documents from different nations suggests widespread evidence-to-policy across the world and calls for a cross-disciplinary approach to addressing loneliness, including efforts to leverage asset-based community development and place-based approaches to tackling loneliness [65].

All 14 countries that published documents on loneliness demonstrated an awareness that loneliness impacted various dimensions including geography (through place of residence and housing, public transport, community services, urban planning), social (some groups are more vulnerable to loneliness than others, social support, technology), health (physical and mental), economics (income, unemployment) or politics (effects of COVID policies, political engagement, working across sectors to address loneliness). Notably, none of the documents reviewed acknowledged that (i) most research on physical health and loneliness is cross-sectional, where the researcher measures both the outcome and the exposures of the study participants at the same time, and thus, the findings of these studies cannot be used to make causal inferences, and (ii) such work does not control for other predictors of health, including, for example, socioeconomic status and actual health conditions. These are important considerations because (a) we cannot be certain that healthy individuals are more likely to get sick if they experience loneliness compared to other healthy individuals who do not experience loneliness, and (b) whether the link between loneliness and health is actually driven by structural inequalities that determine our physical and social environments. We have also found that the documents rarely mention the transient nature of loneliness and the discourse often seems to frame loneliness like an illness that can be treated. The documents also did not address the cultural context (i.e. beliefs, values, religion) that can shape expectations of relationships and the welfare regime.

### Policy targets proposed in the documents

Most countries in our sample showed some attempt at raising public awareness about loneliness (AL, AU, CA, DK, DE, IE, IT, MT, NL, CH, UK, US). Such policies are often informative, but there appeared to be a lack of deadlines and appropriate funding. That means the strategy cannot be evaluated. Another point of concern is the perception that loneliness is something that only affects older adults. Some documents lacked information about how to address loneliness, probably because here is limited evidence of what works and for whom. Also absent was a commitment to evaluation of interventions, which is crucial to verify the effects of any intervention and any risks related to action.

### Recommendations for policy makers

Despite the adoption of an evidence-to-policy approach to loneliness, given the issues noted above, we encourage policymakers to be cautious in making claims in relation to loneliness, and to ensure that part of their strategy includes the funding of research that fill the gaps in knowledge. Policymakers should also ensure that the work they quote includes study populations that are well-represented in all relevant demographics and that the research is able to make causal claims about how loneliness impacts health. The World Health Organisation (WHO) and the European Union have identified the limits of their own knowledge and skills in this field, commissioning experts to write evidence gap reports [66, 67] or GB and DK for example have had loneliness researchers help write their vision and strategy.

Policymakers should also adopt a similar approach in relation to interventions that address loneliness. A recent meta-analytic review [68] suggested that in order for interventions designed to reduce loneliness to be effective, matching the intervention to the loneliness type is essential, whereas a one size fits all will not be effective. For example, social support interventions and social and emotional skills training are all promising interventions for reducing loneliness, albeit they are usually only appropriate for loneliness that is linked to the perceived absence of a close friend or partner and perceived lack social encounters and acquaintances respectively. Such an understanding of the nuances surrounding loneliness interventions is absent from the documents we evaluated, and policymakers will want to fill that gap in their knowledge so that appropriate decisions about intervention work, and suitable funding, can be provided. The effects of current interventions have been shown to be only moderate, highlighting the need for funding for rigorous and systematically developed interventions that are also appropriately evaluated.

Based on our scoping review and underlying evidence we propose a list of actionable recommendations for national and regional governments wishing to establish or incorporate loneliness into their policy documents (Table 3). In sum, we believe that revisiting previous national and local campaigns to identify connection points for loneliness interventions is an effective way to include loneliness into the policy agenda. For example, a walkability campaign that focuses on making cities more pedestrian friendly will benefit individuals in terms of physical health and mental health but it also increases the likelihood of social encounters when walkability is higher [69]. We also believe that sharing best-practice approaches internationally and accessible to everyone ensures the development of a strong knowledge base. The EU has taken the lead as the first supranational union to address loneliness amongst its member states by recently organizing various roundtables and conferences around loneliness [70]. Globally, WHO has recently published an evidence gap report on in-person interventions for reducing social isolation and loneliness [67]. Lastly, we argue that policies would be meaningless if there are no concrete funding streams allocated towards evidence generation, intervention design and implementation and the evaluation thereof. Because our review could not identify clear funding streams for all countries, we strongly encourage policy makers to make the funding streams transparent within their loneliness policies.

**Table 3:** Actionable insights and recommendations for the consideration of policymakers

Category	Recommendation	Implications
**Language**	Use accessible language in policy documents and briefs	To ensure that ideas and concepts can be traced back to the scientific literature, especially because policy makers change over time and newer colleagues may want to revisit previous work for a new campaign.
**Prioritising interventions**	Prioritise interventions according to available funding following the Danish Action Plan [43]	Identify interventions that: (1) can be initiated with the funds that are allocated to the loneliness strategy (2) can be initiated within existing financial frameworks (3) should be initiated and require additional funding.
**Work with funding bodies**	Engage with funding bodies to ensure loneliness remains as a topic of relevance on the agenda	This collaboration can also influence how the funding priorities are laid out in the future. Additionally, collaborating with funding agencies can serve as a reminder of available funding opportunities and how to priorities interventions.
**Revisiting previous campaigns**	Revisit previous national and local campaigns to identify connection points for loneliness interventions	Cost and time effective way to include loneliness into the policy agenda. E.g. revisit campaigns on healthy aging, community development, welfare development, sustainable development, walkability, mental health.
**Sharing best practice across borders**	Promote cross-national collaboration to embed best-practice approaches	A cost-effective way to develop a strong knowledge base on which nations or regional governments around the world can build their context-specific loneliness strategies.
**Setting out the vision**	Have both a strategy to manage loneliness, and an action plan that details need for deployment of effective interventions (e.g. GB & DK)	This distinction helps build and sustain momentum on intervention strategies. Having a national suggestion on evidence-based interventions (or being open about which interventions are backed by evidence and which are not) may help local authorities to decide what kind of interventions best suit their context.
**Evaluation of interventions**	Local authorities should engage with central government when implementing and testing interventions that are not yet evidence-based	Evaluation is not always part of an intervention implementation yet it is essential to ensure that resources are not wasted by implementing ineffective interventions as there are still many knowledge gaps on the effectiveness of loneliness interventions. There should be funding allocated to evaluation of interventions.
**Scalability of intervention**	Decide if your planned intervention will need to be scalable or not and plan finances accordingly	Online loneliness interventions, such as described by [71], are more cost-effective to scale-up than in-person or community level interventions. Overall, there is a lack of evidence on scalable interventions for the general population and at-risk groups of loneliness [72].

### Limitations

The primary limitation of our scoping review was concerned with identifying documents from countries that did not provide information in English. That limitation was partially overcome by the use of Google’s website translator. Another limitation is the reliance on machine translation for the identified documents. Documents were translated into English from German, Danish, Finnish, French, Dutch and Norwegian using DeepL. For German and French, the quality of the translation was checked by the author team and considered sufficient to meet our study aim. The cross-sectional design of our scoping review also does not account for how a country’s policy may have changed over time. This is a general issue in policy evaluation. That limitation can be overcome by conducting this review every two to four years. Another challenge with our study is that the data reflect the existence of policies and not the effectiveness of their implementation. Further, only funding that was explicitly allocated to reducing loneliness and social isolation was considered. We acknowledge that other initiatives that received governmental funding pledges, such as establishing community centres for older adults, might also reduce feelings of loneliness. However, it is beyond the scope of the current paper to identify which initiatives specifically reduce loneliness and how much funding has been allocated to them, especially as evidence on which interventions have proven successful are scarce. Additionally, there may be other funding streams we are not aware of or that might have been part of other documents (e.g., state budgets) not included in this analysis.

More work is needed to assess if the various proposed interventions are implemented and successful. Evaluating interventions is crucial if we want to effectively use the pledged funding, to identify what tools (online or other) are being developed to promote loneliness interventions on national and regional levels and to map out the role of the emerging national loneliness networks.

## Conclusion

Our study provides the first comprehensive overview of the national loneliness policy landscape across 52 countries, highlighting the increasing prioritisation of loneliness and social isolation as significant public health and societal issues. While the momentum in addressing loneliness is evident, with most policies being informed by scientific evidence, gaps remain, particularly around intervention strategies and their effectiveness. Our findings urge policymakers to not only sustain this momentum but to also strengthen their strategies by incorporating rigorous, evidence-based intervention evaluations and fostering international collaborations for knowledge sharing. This approach can enhance the understanding and addressing of loneliness, ensuring interventions are well-targeted, effective, and scalable. By addressing these issues, policymakers can more effectively manage loneliness by directing funds to develop and implement interventions that impact the individual (e.g. through therapy or befriending services, thereby improving public health outcomes) and the community and society by making them genuinely inclusive, thereby increasing social cohesion.

### Supplementary Information

**Supplementary Material 1.**

**Supplementary Material 2.**

## Data Availability

The references to the documents supporting the conclusions of this article are provided in Table 1. Should a link have expired, contact the corresponding author for a pdf version of the translated and original document in question.
