# Supplementary material for: Addressing loneliness and social isolation in 52 countries: a scoping review of National policies
Source: BMC Public Health. 2024 May 1;24:1207. doi: 10.1186/s12889-024-18370-8 (PMC11061917; doi:10.1186/s12889-024-18370-8)
Supplement: Supplementary file 2 — Supplementary Material 2. [file 12889_2024_18370_MOESM2_ESM.docx]

**Supplementary Material**

Table A provides a detailed overview of how each document positions loneliness and social isolation in the geographic, social, health, economic, and political dimensions. This includes associated factors that have been identified in each domain (e.g. protective and risk factors for loneliness; consequences of loneliness). While this table does not directly focus on suggested recommendations and interventions, at times these do overlap in some documents.

**Table A: Detailed overview of geographic, social, health, economic and political dimensions of loneliness and social isolation,** (NR=not reported)

| **Document title (country code)** | **Geographic Domain** | **Social Domain** | **Health Domain** | **Economic Domain** | **Political Domain** |
| --- | --- | --- | --- | --- | --- |
| **The National Action Plan on Aging 2020-2024 (AL)** | - Increased public transport usage by the elderly significantly enhances their social integration. | - Lack of contact with family & friends links to greater loneliness - Low mobility rates & transportation issues links to greater loneliness - Lack of work links to greater loneliness | - Higher levels of chronic diseases & loss of abilities in the elderly compared to other age groups. - Higher levels of chronic diseases, depression, aches, & pains, & decreased mobility in women. - Individuals who suffer from more than one disease & need multiple treatments | - Economic poverty caused by insufficient income for the elderly. - The pension system, despite reforms, faces challenges related to demography & the economy. | - Being denied many rights |
| **Social Isolation & Loneliness**  **Australia (AU)** | - People in Victoria & New South Wales reported higher levels of loneliness compared to the rest of Australia. - There are minimal differences in loneliness levels among Australians living in urban, regional, & rural areas but young men residing in regional areas experience higher rates of loneliness than those in major cities. - Single person households are prone to social isolation. | - Living alone, not being in a relationship (especially recently separated men), & single parenthood were mentioned in relation to loneliness. - Unemployment & receiving income support as factors associated with loneliness. - Social media & its potential impact on loneliness. | - Depression & anxiety as consequences of loneliness. - Premature death as a consequence of loneliness. | - Unemployment, receiving income support, & dissatisfaction with financial situation are factors contributing to the development of loneliness. - Men aged 25–44 on high incomes are more likely to experience loneliness, while women of all ages on low incomes are significantly more susceptible to loneliness compared to women on high incomes. | - Effects of lockdown policies mentioned as contributing to loneliness. |
| **A National Strategy to Address Loneliness**  **Australia (AU)** | NR | - The social impact of workplaces on individuals mentioned. | - Mental health linked to loneliness. | - Health service utilisation costs as an economic burden associated with loneliness, particularly utilisation of mental health services. | NR |
| **Understanding & Defining Loneliness & Social Isolation Australia (AU)** | NR | NR | NR | NR | NR |
| **Overcoming Loneliness**  **Austria (AT)** | - Migration or flight linked to patterns of loneliness. | - Social events, such as a pandemic, can have a profound impact on individuals & communities, affecting mental well-being & social interactions. - The lack of support or opportunities in society, communities, or the workplace can contribute to feelings of isolation & hinder social integration. | - Illnesses & accidents are factors that can contribute to social isolation. - Consequences of loneliness include an increased risk of depression & suicidality, anxiety disorders, dementia or reduced cognitive abilities, heart attack & stroke, & physical signs of chronic stress. | - Unemployment mentioned as a risk factor for loneliness & social isolation. | NR |
| **Report on the Social Isolation of Seniors**  **Canada (CA)** | - Factors contributing to social isolation for seniors include lacking access to transportation, transportation affordability, living alone, work migration & the location of residence (urban/rural). - Additional contributors to social isolation include the lack of awareness or access to community services & programs, insufficient affordable & suitable housing & care options to meet older adults' varied needs, & a loss of the sense of community. - Late onset or age-related disabling conditions such as incontinence or fear of falling when going to & from venues can also increase the risk of social isolation for seniors. - The built environment itself can be a barrier to social participation for seniors, with resistance to activities like walks or community engagement due to physical environment limitations. - Limited access to public washrooms, particularly for those with incontinence, can further inhibit social integration. Accessible public washrooms could serve to enhance social engagement for seniors. | - Elder abuse is a significant concern contributing to social isolation among older adults. - Having no children or contact with family & the changing family structures are factors that can lead to social isolation. - The lack of awareness or access to community services & programs, fear, stigma, ageist attitudes, loss of sense of community, technology, & life transitions can also contribute to social isolation among older adults. - Cultural barriers, such as family responsibilities of some immigrant seniors, can increase the risk of social isolation among older immigrants to Canada. For example, older immigrants caring for grandchildren might have limited time to integrate culturally, learn a language, or participate in community life. - Language barriers can also be a significant factor contributing to social isolation among older immigrants. | - Socially isolated individuals are more at risk of negative health behaviours. - Disabilities can further marginalise individuals, particularly in terms of social inclusion. - Chronic illnesses & social isolation are interconnected, affecting individuals' psychological & cognitive health. | - Low income linked to social isolation. - Lack of affordable & suitable housing & care options linked to social isolation. | NR |
| **A profile of Social Isolation in Canada**  **(CA)** | - Urban dwelling linked to social isolation. | - Changing access to social resources as a method of combating social isolation. - Gender, widowhood or divorce, culture, education, income, & health all linked to loneliness. - Social networks as important protective factors. - Recent immigration, retirement, loss of daily work-related contacts, death of family members or friends, or changes in residence, particularly in older age all linked to loneliness. - Age, education, & urban residence all play a role. | - Decline in physical mobility linked to social isolation with age. - Disability linked to social isolation. - Chronic conditions & illnesses lead to greater social isolation. - Social isolation can have detrimental effects on both physical & emotional well-being, including depression, poor nutrition, decreased immunity, anxiety, fatigue, & social stigma. | - Living in poverty linked to social isolation. | NR |
| **Social Inclusion Strategy 2021-2030**  **(CZ)** | - Active acquisition & distribution of subsidy funds for community work are essential tools for promoting social inclusion in rural areas. - Insufficient or complicated access to housing as one of the basic causes of social exclusion. | - Foreigners in the Czech Republic may be at greater risk of loneliness. - Single parents with a criminal record may be at greater risk of loneliness. | - People with disabilities & mental health problems, along with those suffering from addiction, are vulnerable to social exclusion. - People suffering from any form of addiction &, for example, the LGBTIQ+ minority are examples of specific sub-groups vulnerable towards loneliness. | - A direct link was found between the level of social exclusion & the poverty of families & level of risk to children | NR |
| **The Danish National Strategy Against Loneliness**  **Denmark (DK)** | - Recommends strengthening homeliness & the framework for relationships & communities in residences & care homes. - Recognises the importance of accessibility & transport for communities in residential & local environments. - Recommends strengthening cooperation between housing associations & the rest of civil society regarding residents' participation & engagement in local activities. - Preventing & combating loneliness by working with municipal local plans, strategic urban planning. - Recommends creating flexible housing solutions & support during life's difficult transitions. | - Creating & maintaining safe, inclusive communities to combat loneliness. - Supporting children & young people's attachment to communities in transitions & changes to combat the risk of loneliness. - Create real access to binding communities for young people without employment or education in particular. | - Consider communities & relationships within the framework & direction of the health & care area. - Equip health & care staff to promote relationships & communities & to spot & act on loneliness. - Strengthen bridge-building for civil society & other actors - especially in system transitions & among high-risk health groups. | - Make relationship building & support a high priority focus area in workplaces. - Make it easier for people in vulnerable & exposed positions to become part of working life - Prevent loneliness among the unemployed through volunteering & communities for the unemployed - Everyone should have access to a well-prepared, flexible, & voluntary transition from working life to retirement. - Create real access to binding communities for young people without employment or education in particular. | - Speaks of the importance of the government concerning a national loneliness strategy & providing funding & the importance of regions & municipalities for implementation - Also mentions other key actors in the fight against loneliness (e.g. research institutions, foundations, all employers, civil society.) |
| **The Danish Action Plan Against Loneliness**  **Denmark (DK)** | See Danish National Strategy. | See Danish National Strategy. | See Danish National Strategy. | See Danish National Strategy. | See Danish National Strategy. |
| **Loneliness- recognising, evaluating & resolutely confronting it.**  **Germany (DE)** | - The level of loneliness in a region is influenced by both population losses & population increases. However, describing the regional distribution of loneliness cannot be simplified solely based on urban-rural differences. - Other factors & complexities contribute to the variations in loneliness within different regions. | - Migration background, single parents, & individuals living alone are vulnerable groups susceptible to social isolation. - Vulnerable periods in life are observed during young adulthood (around 18-29 years) & old age (around 80 years & older). These life stages are more prone to experiences of social isolation. | - Mental or physical health impairment linked to loneliness. - The pandemic may have exacerbated loneliness. | - Low income & unemployed individuals at greater risk of loneliness. | - Loneliness has significant political relevance as it correlates with decreased political engagement. - It is imperative to implement political measures at the federal level to address loneliness. |
| **Stronger Together- The HSE Mental Health Promotion Plan 2022-2027**  **Ireland (IE)** | NR | - Social prescribing recognises health's strong correlation with social factors like poverty, isolation, & loneliness. | NR | - Loneliness & lack of social support can lead to reduced community participation & contribution which negatively impacts employment prospects & hinder progress in the workplace. - Reduced productivity, lower job satisfaction, increased absenteeism due to stress or health issues, & longer recovery times may result from loneliness. | NR |
| **Policies for Active Ageing in Italy: What are the Possible Objectives?**  **Italy (IT)** | - To promote well-being & social cohesion, urban planning should consider issues like a sense of belonging, proximity to public services, & quality of life while combating loneliness. - Extending the concept of a sustainable city to suburbs, rural villages, & inland areas can address isolation & loneliness. - Creating healthy & active movement paths to encourage walking groups can combat loneliness effectively. - Supporting housing policies, such as RegioNRl Law 16/2021, aimed at combating loneliness, is crucial (based on article 5 of the law proposal). | - Leveraging the third sector as a valuable resource in co-designing services can establish a robust protection network to address the exacerbated situations of fragility, loneliness, & hardship caused by the pandemic crisis. | - Social exclusion is a significant risk factor negatively impacting the health of older people, affecting both mental & physical well-being. - Policy development should focus on restoring a social function to older individuals to promote their overall health & well-being. | - Allocating more resources to combat poverty & address the loneliness of older people is essential for enhancing their well-being & overall quality of life. | NR |
| **National Strategic Policy for Active Ageing**  **Malta (MT)** | - Older residents in care homes & long-term care facilities are at a higher risk of experiencing loneliness. | - Older age, migration status, illiteracy, & widowhood cause higher risk of loneliness. | - Frailty, a diagnosis of dementia, & other chronic conditions, both physical & mental, increase the risk of loneliness among older people. - Older individuals experiencing reduced mobility, chronic pain, frailty, or other health problems that necessitate long-term care are also more susceptible to loneliness. | NR | NR |
| **One against loneliness. action programme 2022-2025. (NL)** | - - Design of the physical living environment in the neighbourhood is important in combatting loneliness. | - - Creating a strong social basis, increasing social initiative, & encouraging voluntary work all recommended as ways to combat loneliness.   - A strong belief in a community rooted approach to loneliness.   - Reducing loneliness is positioned as a task for society as a whole. | - - Mental health linked to loneliness.   - Working on health prevention approach, includes loneliness & social isolation. | - - Combating poverty to combat loneliness.   - Combating job losses to combat loneliness. | - - Less participation in society leads to loneliness. |
| **Social Resources as Health Protection: Mode of Action & Dissemination in the Swiss Population & in Europe Switzerland (CH)** | NR | - Single parents more at risk of loneliness. - Elderly people, especially elderly women & those living alone at greater risk of loneliness. - Young men at risk of loneliness. - Single individuals, particularly men without a partner, at risk of loneliness. - People with low levels of school education & limited financial resources at risk of loneliness. - Migrants with low socio-economic status, including education & wealth at risk of loneliness. | - Age is linked to loneliness. - Physical illness is a risk factor for loneliness. - Mental illness is a risk factor for loneliness. | - Loss of job as a risk factor for loneliness. - Few financial resources linked to greater loneliness. | NR |
| **Social Resources-Promotion of social resources as an important contribution to mental health. Health & a high quality of life Switzerland (CH)** | NR | The following factors can contribute to loneliness among different age groups:  Children & Adolescents:   - Bullying faced by children at schools & the overall class climate can influence their social experiences & affect their vulnerability to loneliness.   Young Adults & Middle-Aged Adults:   - Social support typically decreases with age, which can lead to feelings of loneliness. - Retirement from work can be a significant life transition that impacts social connections & may contribute to loneliness. - Certain personality traits, such as communication & social skills, can influence individuals' ability to build & maintain social relationships.   Older Adults:   - Older people may experience loneliness due to the loss of loved ones & social connections over time. - Living alone, especially for older men without a partner, can increase the risk of loneliness. - Single parents may also face feelings of loneliness, particularly when balancing parental responsibilities & social needs. | - Chronic illnesses linked to loneliness. - Mental illness linked to loneliness. - Impairments in mobility, hearing & sight linked to loneliness. | - Low financial means linked to loneliness. | NR |
| **Emerging Together: the Tackling Loneliness Network Action Plan**  **UK (GB)** | - The Loneliness Network focuses on "Local & place-based approaches to tackling loneliness." - The Ministry of Housing, Communities, & Local Government (MHCLG) will consider the planning system's role in reducing loneliness. - Local councils play a central role in addressing loneliness by supporting local transport routes & voluntary groups, among other initiatives. | - Level up volunteering infrastructure. - Simplify routes into volunteering, including for people experiencing loneliness, by removing obstacles. - Collaborate with the voluntary sector to leverage government links & work together on volunteering policy. | NR | NR | NR |
| **A Connected Society. A strategy for tackling loneliness**  **UK (GB)** | - Moving to a new town can lead to feelings of loneliness. - The infrastructure in the local area, such as transport links, plays a role in supporting social networks & can aid in reducing loneliness. | - Social & cultural influences can impact the prevalence of loneliness. - Cultural attitudes that stigmatise loneliness may discourage individuals from seeking support & connection. - Changes in ways of working, such as the advent of technology, can limit face-to-face interactions & contribute to social isolation | - Poor health, long-term illness, or disability are associated with loneliness. | - Unemployment linked to loneliness. - Flexible & inclusive volunteering opportunities will help combat loneliness. - The government is working with jobcentres to understand the link between unemployment & loneliness better. | NR |
| **Wellbeing in North Ireland 2021/22**  **UK (GB)** | - People living in urban areas reported significantly higher proportions of loneliness compared to those in rural areas. - In comparison to the Northern Ireland average, those residing in Antrim & Newtownabbey reported significantly lower proportions of loneliness, while individuals in Belfast reported significantly higher levels of loneliness. - People in Belfast reported higher proportions of loneliness than those in Antrim & Newtownabbey, Mid & East Antrim, Mid Ulster, Newry Mourne & Down, & Ards & North Down. - People in Armagh, Banbridge, & Craigavon reported higher proportions of loneliness than those in Antrim & Newtownabbey, Mid & East Antrim, Mid Ulster, & Ards & North Down. - People in Causeway Coast & Glens reported higher proportions of loneliness than those in Antrim & Newtownabbey & Ards & North Down. | - Factors influencing loneliness & social isolation include marital status, religion, dependents, disability, employment status, qualifications, tenure, household count, household internet access, access to a car, deprivation, & local government district. | - Loneliness depends on sex & age. | NR | NR |
| **Social Isolation & Loneliness in Older Adults: Opportunities for the Health Care System 2020**  **(US)** | - The type of housing, whether a private residence, an apartment, or a room in a retirement community or nursing home, can impact an individual's social interactions & feelings of loneliness. - Co-housing or living groups - Retirement communities – recommended only when it is voluntary move - Residential care & nursing homes (yet acknowledges that in some cases this may increase isolation if they are away from family & loved ones; temporary roommates etc does not allow them to forge long-lasting bonds) | - Social relationships with family members, friends, caretakers, spouse, & neighbours can influence feelings of loneliness. - Unfulfilling relationships can contribute to increased loneliness. - Bereavements, such as the loss of loved ones, can lead to heightened feelings of loneliness. - Gay, lesbian, & bisexual individuals may face unique challenges in social connections, potentially impacting their experiences of loneliness. - Social prescribing & peer support groups suggested to combat loneliness. | - Recognises the public health impact of social isolation & loneliness. - Health care systems should create opportunities for clinicians to partner with researchers to evaluate the application of currently available evidence-based tools to assess social isolation & loneliness in clinical settings, including testing & applications for specific populations. - Social isolation should be included in the electronic health record or medical record. - Health professions schools & colleges as well as direct care worker training programs should include education & training related to social isolation & loneliness in their curricula, optimally as interprofessional team-based learning experiences. - Health professional associations should incorporate information about the health & medical impacts of social isolation & loneliness on older adults in their advocacy, practice, & education initiatives. - Health care providers, organizations, & systems should partner with social service providers, including those serving vulnerable communities, in order to create effective team-based care (which includes services such as transportation & housing support) & to promote the use of tailored community-based services to address social isolation & loneliness in older adults. - CBT, interpersonal psychotherapy, & mindfulness recommended as mental health interventions | NR | NR |
| **Addressing Social Isolation to Improve the Health of Older Adults: A Rapid Review**  **(US)** | NR | NR | - Physical activity interventions show the most promise at improving the health of older adults - Health systems should rigorously evaluate their efforts to increase the evidence base & share results with other health care systems. | NR | NR |
| **Our Epidemic of Loneliness and Isolation. The U.S. Surgeon General’s Advisory on the Healing Effects of Social Connection and Community (US)** | - Design the built environment to promote social connection - strengthen social infrastructures: programs (such as volunteer organizations, sports groups, religious groups, and member associations), policies (like public transportation, housing, and education), and physical elements of a community (such as libraries, parks, green spaces, and playgrounds) that support the development of social connection. | - Actively seek and build partnerships with other community institutions (schools, health organizations, workplaces) to support those experiencing loneliness and social isolation, and to create a culture of connection in the broader community. - Establish and scale community connection programs - Invest in local institutions that bring people together | - Loneliness and social isolation increase the risk for premature death - Train health care providers on the risks associated with social disconnection (e.g., isolation, loneliness) - Expand public health surveillance and interventions - Insurance companies should provide adequate reimbursement for time spent assessing and addressing concerns about social disconnection (e.g., isolation, loneliness), and incorporate these measurements into value-based payment models. | - The lack of social connection can have significant economic costs to individuals, communities, and society. - Social isolation among older adults alone accounts for an estimated $6.7 billion in excess Medicare spending annually, largely due to increased hospital and nursing facility spending. - Stress-related absenteeism attributed to loneliness costs employers an estimated $154 billion annually. | - Adopt a “Connection-in-All-Policies” approach - Advance policies that minimize harm from disconnection - Establish cross-departmental leadership at all levels of government |
